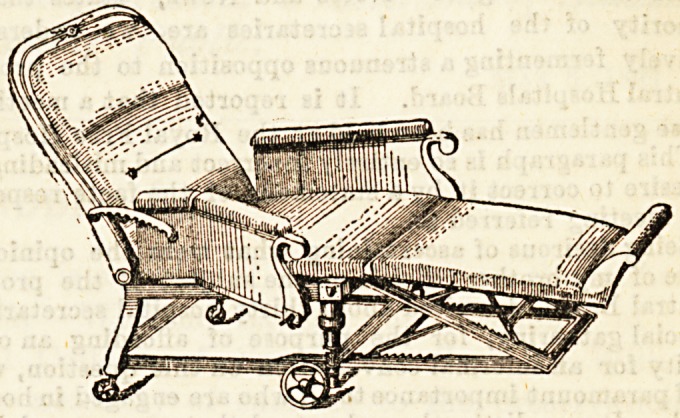# Invalid Chairs, II

**Published:** 1893-04-29

**Authors:** 


### PRACTICAL DEPARTMENTS.

### INVALID CHAIRS.—II.

Last week we gave some description of bath and carrying chairs, in which there are now so many improvements; and these improvement are not lacking when we come to consider those chairs and couches adapted for use in the sick-room.

Of these there are an ever-increasing number, mechanical skill and scientific knowledge combining to meet the requirements of all cases of illness and accident, so that the greatest possible comfort may be secured to the invalid.

We are most of us familiar with the Ilkley couch, an nvention which has been an immense boon to thousands. Perhaps only those who have been compelled to spend any length of time in a recumbent position can fully appreciate its many advantages over the most comfortable of ordinary sofas. Messrs. Leveson, 90, New Oxford Street, have many elaborated varieties of this old friend to show. One of these differs from the usual make in that instead of having a loose mattress, it is supplied with a luxurious spring stuffing, which somewhat improves its appearance, though to our ideas the iadvantages of the loose mattress are superior, particularly for the conveniences of travelling, as with the mattress removed the couch will fold up into a comparatively small compass. Both ends can be raised or low ered to suit the comfort of the patient, by means of a wooden rack, and the centre part can also be raised in the same manner, this position being a specially comfortable one. The change of posture thus obtained is very grateful to any one condemned to spend weary days on a sofa. A great recommendation of these couches is that their price brings them within the reach of all. They can be had complete, with a cretonne-covered mattress, made of strong birch, caned, an d mounted on castors, from three guineas and a-half, a really modest sum for so delightful a piece of furniture. More elaborate upholstery can, of course, be supplied if desired, and the price increases in proportion to the little improvements introduced.

Messrs. Leveson are also the makers of a mechanical couch, suitable alike for use by day or night. This, like the Ilkley, can be altered to several positions, the centre being raised or lowered by the turning of a handle in the front. There is also a scroll foot-rest, a considerable addition to the comfort of the patient.

A new design for chair and couch combined has been brought out by this firm. As a couch it "can be used at its full length, and the back, centre, or foot part raised to any inclination, and it can be easily closed by a simple telescopic action, and becomes a chair with graduating back." One of its great advantages is the ease with which it can be folded up for travelling, the legs being quickly unscrewed by the hand.

Our illustration shows a reclining chair which boasts of a special feature in the opening sides, which are hinged, and thus greatly facilitate the assisting of a helpless invalid in and out. This can also be turned into couch or chair at pleasure, the foot support being adjustable, and can be stowed right away under the seat. The spring graduating back can be regulated by a movement of the brass quadrant at the side. When closed up the chair has the appearance of a delightful smoking chair, not at all that of a piece of sick-room furniture.

Some very cleverly contrived automatic invalid couches have been recently designed by various makers. Of these we shall hope to be able to make special mention on some uture occasion.

The construction of spinal carriages has been also very much perfected, and Messrs. Leveson have a special design which would seem to have all the conditions required to give the patient the maximum amount of rest. It is light in make, there being no more solid woodwork about it than is absolutely necessary, mounted on Cee springs, and fitted with an inside frame enabling the patient to be lifted in and out with no disturbance of position. This frame is supplied with a graduating back rest, making it possible for the head and shoulders to be raised when desired.

An inspection of these additions to the comfort of the sick leaves us with the impression that no pains are spared in their design, and that their construction is carried out on the best principles.

## Figures and Tables

**Figure f1:**